# Evaluating Human-Robot Interaction With a Socially Assistive Robot in Pediatric Health Care: Mixed Methods Pilot Study

**DOI:** 10.2196/95702

**Published:** 2026-11-06

**Authors:** Janika Leoste, Slavko Rakić, Kristel Marmor, Kadi Krinal, Liina-Harjus Soostar, Katariina Maribel Vink, Andres Käver, Katrin Aasma, Katie Beatty, Leo Benjamin Leoste

**Affiliations:** 1 IT College, Tallinn University of Technology, Ehitajate Tee 5, Tallinn, Harjumaa, 19086, Estonia,; 2 School of Educational Sciences, Tallinn University, Tallinn, Harjumaa, Estonia; 3 Faculty of Technical Sciences, University of Novi Sad, Novi Sad, Serbia; 4 Centre for Digital Health, Department of Health Technologies, Tallinn University of Technology, Tallinn, Harjumaa, Estonia; 5 Tallinn Children's Hospital, Tallinn, Harjumaa, Estonia; 6 The Ohio State University, Ohio, OH, United States

**Keywords:** socially assistive robots, human-robot interaction, hospital navigation, mixed methods, technology acceptance

## Abstract

**Background:**

Socially assistive robots are increasingly explored in health care, but evidence from routine pediatric outpatient settings remains limited.

**Objective:**

This study examined how contextual support and human facilitation shaped visitor interaction with the Temi V3 robot and which sociotechnical conditions were associated with practical acceptance according to staff.

**Methods:**

A mixed methods pilot combined structured behavioral observation across baseline, poster support, and staff facilitation conditions with 4 staff focus group interviews. The observation unit was a visitor passage through the lobby zone; the qualitative sample comprised 12 staff members with direct exposure to the robot. Quantitative indicators were analyzed descriptively, and staff interviews underwent deductive-inductive qualitative content analysis.

**Results:**

In total, 675 visitor passages (children: n=398, 59% and adults: n=277, 41%) were recorded (baseline: n=257; poster support: n=160; and staff facilitation: n=258). Looking at the robot increased from 8 (3.1%) passages at baseline to 30 (18.8%) with poster support and 80 (31%) with staff facilitation. Screen or app use increased from 2 (0.8%) passages at baseline to 4 (2.5%) with poster support and 14 (5.4%) with staff facilitation. Observer notes indicated stronger visible responsiveness among children, although age category was not linked to individual behavior codes. Staff emphasized role clarity, child and family value, human mediation, functional reliability, multilingual content, and organizational ownership.

**Conclusions:**

Observed visitor interaction was higher with poster support and staff facilitation than at baseline. These descriptive findings and staff perceptions informed a provisional 4-domain framework comprising role clarity, interaction affordance, social mediation, and organizational embedding. The framework identifies potential implementation priorities; visitors’ own experiences and acceptance require direct assessment.

## Introduction

### Background

Socially assistive robots (SARs) are designed to support people through social interaction without providing direct physical assistance [1,2]. In health care, they have been explored as guides, companions, educators, telepresence platforms, and communication mediators [3-5]. Hospitals are especially relevant settings because they combine logistical complexity with emotional strain, repeated information needs, and heavy staff workload [3,6,7].

Pediatric outpatient environments add a further layer of complexity. Children often face uncertainty, waiting, unfamiliar spaces, and procedure-related stress, while parents must simultaneously manage navigation, appointments, and the child’s emotional state. Prior reviews suggest that social robots can support children’s well-being during medical treatment through distraction, engagement, and emotionally lighter interaction, but the evidence remains heterogeneous and strongly context dependent [8,9]. Hospital human-robot interaction (HRI) reviews likewise show that pediatric settings are promising, while many studies remain short-term, small-scale, or conducted under controlled conditions instead of routine clinical conditions [3,7].

Implementation is an additional challenge. Health care robot adoption is rarely determined by technical capability alone. Acceptance depends on whether users understand what the robot is for; whether it fits work processes; whether it is easy to use; and whether organizations provide the training, maintenance, and responsibility structures needed for sustained use [10-12]. Recent European survey evidence also suggests that public support for SARs in health care is conditional. People appear more comfortable with bounded assistive roles, such as information provision and guidance, than with higher-stakes gatekeeping or quasi-clinical decision roles, and privacy remains a recurring concern [13].

### Study Context

The present study examined a Temi V3 robot in the second-floor outpatient area of Tallinn Children’s Hospital in Tallinn, Estonia. The Temi V3 is a mobile platform with screen-based interaction, speech output, and navigation capabilities [14]. The robot was introduced as a public-facing assistive technology for wayfinding, simple informational support, and child-friendly engagement during waiting. Related earlier work by the research team examined scenarios for telepresence robots in health care [15]; the present manuscript reports a separate 2025 field pilot focused on a SAR in a pediatric outpatient setting.

### Theoretical Background

#### SARs in Hospitals and Pediatric Care

The health care robotics literature includes several overlapping categories, including service robots, social robots, SARs, companion robots, and telepresence robots [2,4,5]. The term SAR is the most relevant term for the robot used in the study (Temi V3), as it was not intended to perform invasive or physically assistive clinical work. Its intended value lay in communication, guidance, and emotionally supportive interaction, which aligns with assistance through social engagement [1].

Systematic reviews show that hospital SARs have been implemented in reception, wayfinding, education, rehabilitation, companionship, telepresence, and administrative support [3,4,7]. Broader surveys similarly suggest that near-term adoption is most plausible in bounded domains such as communication, navigation, logistics, telepresence, and patient-facing service functions, where robots can add value without assuming clinical agency [6]. Many hospital robots therefore occupy an intermediate position: they are neither entertainment devices nor autonomous care professionals, but hybrid systems whose usefulness depends on how well they extend existing human service processes.

In pediatric care, published work has frequently focused on play, distraction, procedure support, emotional comfort, and engagement during waiting or treatment. Moerman et al [8] concluded that social robots may support children’s well-being, especially by reducing distress and stimulating positive engagement, while also noting that much of the evidence comes from small or highly contextual studies. Kabacińska et al [9] found that children, parents, and health care providers may all see value in pediatric hospital robots, while emphasizing workflow fit, co-design, and responsible implementation.

The field nevertheless remains uneven. Reviews report positive interaction outcomes alongside recurring friction points, including technical unreliability, overestimated autonomy, narrow interaction repertoires, poor personalization, and weak integration into the social context of care [5,7,10]. A robot’s observed use in a hospital cannot therefore be understood as a property of the machine alone. It also reflects the information environment, user expectations, staff mediation, physical setting, and local routines.

#### Acceptance and Implementation of Health Care Robots

Technology acceptance research offers a useful starting point. The technology acceptance model (TAM) identifies perceived usefulness and perceived ease of use as central drivers of adoption [16]. The UTAUT (unified theory of acceptance and use of technology) adds social influence and facilitating conditions, which are particularly relevant when technologies are introduced through organizational channels [17]. Staff and visitors assess technical capability together with usefulness, understandability, approachability, and visible social endorsement.

For social robots, the Almere model extends acceptance thinking by incorporating trust, enjoyment, social presence, and anxiety [18]. Although developed in work with older adults, its broader insight is relevant here: acceptance of a social agent reflects both functional evaluation and the felt quality of interaction. In pediatric contexts, playful engagement, attention redirection, and perceived warmth may matter alongside instrumental usefulness [8,9].

Acceptance models alone do not capture implementation fully. Reviews of SARs in health and social care identify insufficient training, unclear role boundaries, additional workload, technical unreliability, privacy and safety concerns, weak customization, and the absence of local champions or maintenance structures as recurring barriers [10,11,19]. Stakeholder-oriented design studies similarly indicate that acceptability improves when robot functions are visibly useful, easy to understand, robust in busy environments, and aligned with actual care needs [11].

Acceptance is also multiactor. Children, parents, frontline nurses, administrative staff, technical staff, and managers may evaluate the same robot differently because they encounter different benefits, burdens, and risks. Early interest may coexist with reservations about accountability, privacy, maintenance, and role boundaries [10,13,19]. Qualitative work with nursing professionals likewise indicates that robots gain legitimacy when attached to concrete support tasks and when organizational conditions for integration are explicit [12,20].

### Analytical Framework and Study Aim

Drawing these strands together, the study used 4 sensitizing domains: role clarity, interaction affordance, social mediation, and organizational embedding. Role clarity concerns whether the robot has an obvious and locally meaningful purpose. Interaction affordance concerns whether visitors and staff can understand how to start, continue, benefit from, and recover from interaction. Social mediation concerns whether signage, demonstration, and visible endorsement reduce uncertainty and invite use. Organizational embedding concerns workflow, training, support, maintenance, content management, and responsibility. The 4 domains were developed as an author synthesis of TAM, UTAUT, the Almere model, and implementation evidence [10,11,16-20]. They guided deductive coding and mixed methods interpretation, while inductive analysis remained open to recurrent issues raised by staff. The framework is provisional and is not presented as a validated scale.

The aim of the study was to examine how contextual support shaped observable visitor interaction with Temi V3 and which sociotechnical conditions staff associated with its practical acceptance in pediatric outpatient care. The following research questions (RQs) guided the study:

- RQ1: How did the 4 observed interaction indicators (looking at the robot, approaching it, touching it, and using the robot’s screen or app) vary across baseline, poster support, and staff facilitation conditions?
- RQ2: Which sociotechnical factors did hospital staff identify as enabling or constraining practical acceptance and integration of the robot?

## Methods

### Research Design and Integration

The study used a mixed methods pilot design with separate quantitative and qualitative analyses and convergent interpretation [21]. The quantitative strand examined spontaneous visitor interaction with the robot under 3 sequential implementation conditions. The qualitative strand explored how staff perceived the robot’s usefulness, limits, and implementation requirements through small focus group interviews. The strands were analyzed separately and then interpreted together to relate observable visitor behavior to staff perspectives on implementation, without treating staff accounts as evidence of visitors’ own perceptions.

This design was selected because unobtrusive observation was needed to document what people actually did near the robot under routine hospital conditions, while interviews were needed to understand how staff interpreted the robot’s role, usefulness, support burden, and fit with everyday work. The study did not link the 2 datasets at the person level. It used them as complementary evidence about immediate visitor uptake and organizational implementation.

The participant groups were intentionally different. The observation strand included naturalistic visitor passages, whereas the qualitative strand included employees with direct experience of using, observing, supporting, or coordinating the robot. Children, parents or guardians, and other adult visitors were not interviewed; claims about these groups are therefore limited to observed behavior and staff perceptions. RQ1 was addressed through condition-level behavior counts, observer-assigned age categories, and contemporaneous field notes, and RQ2 through staff interviews. Integration occurred after the separate analyses.

### Setting and Robot

The study took place in the second-floor outpatient lobby at Tallinn Children’s Hospital. Families in this area navigate several specialties and may encounter uncertainty about room locations, changing numbering systems, and waiting routines. The robot’s intended public-facing functions included wayfinding, basic hospital information, and child-friendly engagement during waiting.

The robot’s pilot interface combined hospital-specific and generic elements in the national language (Estonian). Mapped hospital destinations and selected hospital information were available alongside generic Temi content, including news and simple entertainment. The robot’s hardware, mapped destinations, screen content, and physical location remained unchanged across the 3 observation conditions. The experiment’s conditions differed only in the external contextual support provided.

### Quantitative Strand: Structured Behavioral Observation

#### Participants, Sampling, and Eligibility

The unit of analysis was a visitor passage through a predefined lobby observation zone during the scheduled observation periods. Consecutive passages were included when a person entered the zone within an observation window. Passages outside the zone or outside the scheduled period were excluded. No further demographic or behavioral eligibility criteria were applied because the purpose was to document naturalistic visitor flow. Observation was kept nonintrusive and anonymous, so repeat passages by the same person could not be identified or excluded. The reported totals represent passages and cannot be interpreted as counts of unique visitors. Observers assigned a descriptive age category of child or adult. Age category was not linked to each individual behavior code in the aggregated dataset. Age-related findings are therefore based on phase-level counts and field notes, and no statistical comparison of child and adult engagement rates was possible.

#### Implementation Conditions and Observation Protocol

Observation occurred under 3 sequential conditions (Figure 1): baseline (May 22‐23, 2025), poster support (June 5‐6, 2025), and staff facilitation (June 12‐13, 2025). At baseline, the robot was available without added contextual support. In the poster condition, a sign introduced the robot’s name, purpose, and simple interaction cues. In the staff facilitation condition, a hospital intern stood near the robot and verbally invited visitors to interact.

Two observers monitored the lobby using a predefined protocol. One focused on entries and exits, while the other recorded visible interaction near the robot. Engagement was operationalized as 4 progressive but separately tallied indicators: looking at the robot, approaching it, touching it, and using the screen or app. A passage could contribute to more than 1 indicator, so the indicators were not mutually exclusive. The 4 indicators were retained as separate outcomes and were not averaged across conditions.

**Figure 1.**
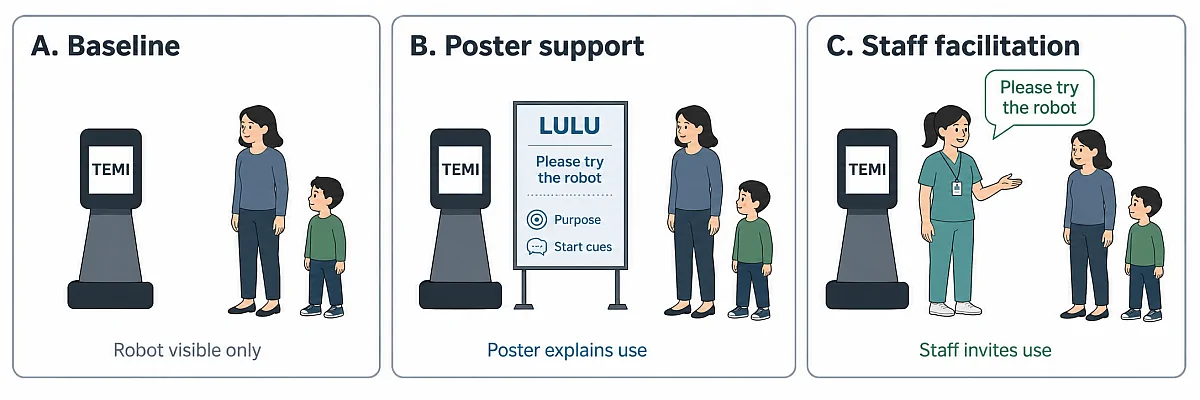
Three sequential implementation conditions in the pediatric outpatient lobby. The robot and its location remained unchanged; only the contextual support differed.

#### Quantitative Analysis

Frequencies and percentages were calculated separately for each behavior indicator and condition. As an additional descriptive summary, the 4 indicator counts were summed within each condition, divided by the number of visitor passages in that condition, and multiplied by 100. This summary represents recorded indicators per 100 passages, not the percentage of passages with any interaction or a measure of acceptance, because the indicators may overlap. The analysis was descriptive because the field conditions were sequential, nonrandomized, and conducted on different dates. No inferential tests or *P* values were calculated. The quantitative results were compared with staff themes during mixed methods interpretation.

### Qualitative Strand: Staff Focus Groups

#### Participants and Sampling

The qualitative strand comprised 12 health care professionals interviewed in 4 small focus group sessions. Sampling was purposive and criterion based. Inclusion required employment at Tallinn Children’s Hospital and direct exposure to the robot through use, observation, support, or deployment-related coordination. The available role information represented frontline clinical and nursing perspectives as well as coordination-level and senior organizational perspectives. Staff without direct robot exposure, hospital visitors, children, and parents or guardians were excluded.

To protect employees from possible deductive identification within the relatively small hospital workforce, detailed demographic data, including exact age, gender, tenure, and job title, were not collected. The manuscript therefore reports only the available broad role-level information. The limited demographic detail is acknowledged as a study limitation, and a structured summary of the available sample characteristics is provided in Multimedia Appendix 1.

#### Interview Procedure and Analysis

The interview guide covered opportunities for robot use, factors supporting and hindering use, suitable functions in interaction with children and parents, and organizational needs connected to implementation. Participation was voluntary. Sessions were audio-recorded, transcribed using automated speech recognition, manually corrected, deidentified, and stored on institutional infrastructure for the approved research purpose.

A deductive-inductive qualitative content analysis was used [22,23]. Deductive coding drew on the interview topics and the 4 sensitizing domains. Inductive coding remained open to recurrent issues raised by participants. Codes and themes were reviewed iteratively in line with established qualitative analysis guidance [24]. The focused sample was considered information rich for exploratory analysis because all participants had direct exposure to the robot and its organizational setting [25]. A supplementary overview of the resulting themes is provided in Multimedia Appendix 1.

#### Mixed Methods Integration

After separate analysis, condition-level observation patterns were compared with staff perceptions of usefulness, interaction difficulty, human mediation, and organizational support. This comparison informed the provisional 4-domain framework presented in the Results section. The datasets involved different participant groups and were not linked at the person level; staff accounts were used to contextualize observed patterns, not to establish visitors’ own perceptions or reasons for interacting.

### Ethical Considerations

The study comprised anonymous, noninvasive observation and voluntary staff interviews. Ethics approval for observation was granted by the Tallinn Children’s Hospital Research Ethics Committee (TCH/2025-01), and the interview component was reviewed by Tallinn University of Technology’s Academic Ethics Research Committee. Because observation involved no recordings or identifiable personal data, individual consent was waived for the public space observation. Staff participants provided informed consent. Interview recordings and transcripts were stored securely, and the manuscript reports only aggregated observations and anonymized themes. All procedures complied with the General Data Protection Regulation (European Union 2016/679).

## Results

### Visitor Engagement Across the 3 Field Conditions

Across the 3 observation phases, 675 visitor passages were recorded, comprising 398 child and 277 adult passages. Each of the 4 separately recorded behavior indicators increased from baseline to poster support and again to staff facilitation (Table 1). The sums of the 4 indicator counts were 14, 52, and 156, respectively, corresponding to 5.4, 32.5, and 60.5 recorded indicators per 100 passages. These descriptive summaries include overlapping behaviors and are not percentages of passages with any interaction.

**Table 1.** Structured observation results by implementation condition, with a descriptive sum of overlapping indicator counts^a^.

Condition	Looked, n (%)	Approached, n (%)	Touched, n (%)	Screen or app use, n (%)	Indicator sum^b^, n (per 100 passages)
Baseline (n=257)^c^	8 (3.1)	2 (0.8)	2 (0.8)	2 (0.8)	14 (5.4)
Poster support (n=160)^d^	30 (18.8)	12 (7.5)	6 (3.8)	4 (2.5)	52 (32.5)
Staff facilitation (n=258)^e^	80 (31.0)	44 (17.1)	18 (7.0)	14 (5.4)	156 (60.5)

a Counts represent visitor passages, not confirmed unique individuals.

b The final column sums the 4 overlapping indicator counts within each condition. Values in parentheses in this column are counts per 100 passages. Because a single passage can contribute to multiple indicators, the sums do not represent distinct interaction episodes, passages with any interaction, or unique visitors.

c Includes 108 child and 149 adult passages.

d Includes 120 child and 40 adult passages.

e Includes 170 child and 88 adult passages.

In the baseline phase (n=257), the robot was largely ignored. Occasional automatic voice output did not translate into sustained use. Looking was recorded for 8 (3.1%) passages, while approaching, touching, and screen or app use were each recorded for 2 (0.8%) passages.

When the poster was added, visible interaction increased (n=160). Looking was recorded for 30 (18.8%) passages, approaching for 12 (7.5%) passages, touching for 6 (3.8%) passages, and screen or app use for 4 (2.5%) passages. The sign provided the robot’s name, purpose, and interaction cues without changing its technical capabilities; whether visitors found these cues clearer or more useful was not assessed.

The highest counts occurred when a staff mediator stood near the robot and explicitly invited use (n=258). Looking was recorded for 80 (31.0%) passages, approaching for 44 (17.1%) passages, touching for 18 (7.0%) passages, and screen or app use for 14 (5.4%) passages. In descriptive terms, the phases represented progressively stronger levels of contextual and social support.

Contemporaneous field notes indicated that children were the most visibly responsive users in each phase, initiating contact through looking, approaching, touching, or play, while adults more often engaged in a supervisory or instrumental manner, for example, by watching a child interact or asking what the robot was for. Because age category was not linked to person-level behavior codes, these observations do not establish age-specific engagement rates and are not presented as a statistical comparison.

### Staff Perspectives on Robot Use and Adoption

#### Staff Participant Background

The 4 focus groups included 12 staff members with direct exposure to the robot and represented both frontline clinical and organizational perspectives. The available role-level information is summarized in Multimedia Appendix 1. The absence of exact age, gender, tenure, and job title counts is considered when interpreting the transferability of the qualitative findings.

The interviews produced 5 interrelated themes. Multimedia Appendix 1 provides a compact overview; the main findings and illustrative quotations are presented below.

#### The Robot Was Accepted When Its Role Was Concrete and Visible

Staff were generally positive about the robot and did not describe strong resistance to its presence. Staff described a concrete and visible function as important for the robot’s acceptance. The most frequently mentioned use case was wayfinding: helping families locate rooms, navigate the outpatient floor, or reduce confusion related to changing numbering systems and unfamiliar layouts. One participant explained:

*If you have two children with you and you are looking for a four-digit room number, that is quite difficult for the parent…for the parent it is easier, for the child it is fun, so it is a win-win.*

[P11]

Another participant described the robot as a practical alternative when staff could not leave their own work area:

*When someone asks me where room A2071 is, I have no idea. Then I say, let’s look at Lulu, and they disappear with the robot. I do not have to run with them myself.*

[P12]

Several participants contrasted this concrete usefulness with a more diffuse sense that the robot was present but not yet integrated. As one nurse put it:

*For me it is actually still a bit unfamiliar.*

[P3]

#### Child Engagement Created Indirect Value for Parents and Staff

Participants consistently described children as drawn to the robot’s movement, voice, and games. Even when parents were initially reserved, children often initiated contact, and parents then followed. One participant described the robot as “something very interesting” for children and added:

*The child’s attention goes there, not, for example, to the procedure.*

[P5]

Another described a similar impression:

*For a moment they forget.*

[P4]

Staff framed the benefit as indirect as well as direct: engaging the child could make waiting easier for parents and reduce some low-level interaction burden for staff. These accounts describe perceived value; the study did not directly measure anxiety, distraction, or satisfaction.

#### Human Mediation and Local Prompting Were Critical

Staff accounts were consistent with the higher interaction counts observed in the staff facilitation condition. Staff said the robot was used more when someone invited families to try it, explained what it could do, or modeled its use. A coordination-level participant stated:

*When there was a person next to it, inviting people to use it, then it was used more.*

[P2]

The same participant identified the practical constraint:

*There was no resource to put anyone next to it to introduce it.*

[P2]

Frontline staff described a similar need from another angle. One nurse noted:

*Medical staff do not guide Lulu use.*

[P3]

Another participant preferred a dedicated introduction:

*There is a specific time, no one interrupts, and then you know exactly.*

[P5]

Staff perceived invitations and demonstrations as helpful for initiating interaction. Visitors’ own reasons for engaging or not engaging were not assessed.

#### Limited Functionality Constrained Broader Acceptance

Although participants valued the idea of the robot, they also identified practical limitations. These included slow movement, charging and troubleshooting problems, unclear audibility, uneven content quality, and the need for multilingual support. One participant summarized the technical issues:

*More speed. Sometimes it does not charge.*

[P4]

Another suggested that the robot display a support contact when problems occurred [P6]. Content was also a recurring issue. A senior participant argued:

*I would remove the news. No one reads the news, children do not want to read them, and parents mostly watch the children play.*

[P12]

The participant proposed short, relevant “tips and tricks for parents” instead. A nurse noted that many visitors were Russian speakers while the device did not support Russian-language interaction [P3]. Positive attitudes therefore did not remove routine usability requirements.

#### Sustainable Use Required Organizational Ownership

A recurring issue was responsibility. Staff did not always know whom to contact when the robot malfunctioned, who could update its content, or who was expected to coordinate its place in daily work. One participant stated:

*We need to choose Lulu’s owner.*

[P12]

They added that if the hospital became the owner, responsibility should be assigned within the hospital. Another participant emphasized staff capacity:

*The main problem here is time resource…people do not do it because they simply do not have the time.*

[P2]

Participants were open to broader use and viewed local responsibility for support, updates, and integration as important to its implementation.

### Integrated Interpretation and Provisional Framework

Observed indicator counts were higher with poster support and highest with staff facilitation. Staff accounts were consistent with the potential importance of clear functions and human prompting but did not establish why visitors interacted or whether they experienced less uncertainty. The strands provide complementary evidence about observable behavior and staff perceptions of implementation, rather than direct confirmation of visitors’ acceptance.

Field notes described children’s visible responsiveness, while staff accounts identified perceived benefits and organizational needs. Together, these findings informed the provisional *mediated acceptance framework* in Table 2. The framework organizes possible links between visitor-facing support and staff-identified implementation conditions; it has not been validated against direct visitor feedback or sustained service outcomes.

**Table 2.** Provisional mediated acceptance framework linking observed visitor behavior with staff perceptions and proposed implementation priorities.

Domain	Evidence in this study	Proposed implementation priority
Role clarity	All 4 recorded indicators were higher with poster support than at baseline; staff emphasized wayfinding and room finding.	Define a bounded service task and make its purpose visible at the point of use
Interaction affordance	Screen or app use remained limited; staff-identified content, language, speed, audibility, charging, and recovery constraints.	Provide site-relevant multilingual content and clear start, use, and recovery cues
Social mediation	All 4 observed indicators were highest with staff facilitation; staff also described prompting and onboarding as necessary.	Consider early facilitation and brief staff onboarding and assess their usefulness directly with visitors and staff
Organizational embedding	Staff requested a named owner, support contact, content process, maintenance, troubleshooting, and protected time.	Assign ownership and integrate support, updates, maintenance, and evaluation into workflow

## Discussion

### Main Findings

This mixed methods pilot examined how a socially assistive robot was received in a pediatric outpatient setting and which conditions shaped practical acceptance. Across all 4 observed indicators (looking at the robot, approaching it, touching it, and using the robot’s screen or app), interaction was lowest when the robot was present without explanation, increased when a poster clarified its role, and was highest when an intern invited and demonstrated use. Staff accounts provided a complementary implementation perspective rather than a direct explanation of visitors’ experiences. Staff evaluated the robot as a service tool whose value depended on a concrete role, relevant and multilingual content, technical support, workflow fit, and visible organizational ownership.

Taken together, observed visitor behavior and staff perceptions informed the provisional mediated acceptance framework. The robot’s hardware, location, and interface did not change across the 3 field phases; the surrounding support did. The results should nevertheless be read as descriptive associations. The sequential naturalistic design does not establish that the poster or facilitator alone caused the observed differences.

### Interpretation in Light of Prior Research

The stepwise increase in recorded behavior can be interpreted in light of technology acceptance theory. Clearer information and human facilitation are possible mechanisms consistent with perceived usefulness, ease of use, social influence, and facilitating conditions [16,17]. These constructs were not measured among visitors, so this interpretation remains theoretical rather than an empirical explanation of their behavior.

The stronger visible responsiveness of children also aligns with pediatric and hospital robotics literature. Earlier reviews suggest that children often engage readily with social robots when interaction is concrete, emotionally legible, and easy to interpret [3,8]. Staff interviewees described movement, voice, games, and distraction during waiting as meaningful for children. Staff evaluated the robot in terms of usefulness, reliability, and support burden, while field notes described adult visitors more often taking supervisory or instrumental roles. These observations and staff accounts do not establish differences in perceptions between children and adult visitors, although they suggest questions relevant to research on the broader care ecosystem [7,9]. The present age-related interpretation remains cautious because it is based on field notes and lacks linked age-by-behavior counts.

The findings also support a sociotechnical interpretation of acceptance. Staff did not ask for a more spectacular or more human-like robot. They asked for clearer functions, stronger content, multilingual capacity, easier troubleshooting, training, and responsibility. These priorities mirror barriers and enablers identified in implementation reviews, where maintenance, workflow fit, reliability, and stewardship influence whether robots move beyond pilot visibility [10,11,19,20]. Staff descriptions of child engagement and usability constraints raise hypotheses relevant to enjoyment, social presence, trust, and ease of use in the Almere model [18]. These constructs were not assessed directly among visitors and should not be treated as observed acceptance outcomes.

The integrated framework adds a practical bridge between these traditions (Table 2). TAMs explain why a person may approach and try the robot, while implementation research explains whether the organization can sustain the service. In this study, observation documented behavior under different support conditions, while staff accounts identified perceived longer-term needs such as content management, troubleshooting, and ownership. The contribution is a provisional synthesis of these complementary perspectives, not evidence of visitors’ perceived usefulness, trust, or acceptance.

### Practical Implications

The descriptive observation patterns and staff accounts suggest that hospitals could evaluate clear signage, explicit interaction cues, and early human facilitation when introducing SARs. These approaches may support visitor interaction, but their effects on visitors’ understanding, uncertainty, and perceived usefulness were not assessed in this study. Staff-identified wayfinding, waiting time engagement, parent guidance, and simple procedural information as potential use cases. These suggestions are consistent with roles discussed in the broader health care robot literature [4,5], but the present observations did not establish successful task completion or benefits perceived by visitors. Such functions should therefore be evaluated with children, parents or guardians, and other adult visitors before broader implementation.

The interviews also suggest that a child-facing function may create indirect value for other people in the care environment. A robot that holds a child’s attention during waiting may make navigation or preparation easier for parents and may reduce some routine interaction demands on staff. Future deployments should assess this potential chain of benefit directly; visible engagement alone is insufficient. Hospitals should therefore define expected outcomes for each use case, such as successful wayfinding, reduced uncertainty, or perceived helpfulness, and select measures that correspond to those outcomes. Staff accounts emphasized the need for organizational anchoring. A hospital should, with the help of relevant stakeholders, define what the robot does, where its task begins and ends, who updates content, who responds to faults, and how value will be assessed. Staff onboarding should include routine use and common recovery situations. The framework in Table 2 summarizes staff-informed priorities for implementation; whether these priorities improve visitor experience or sustained use requires further evaluation.

### Limitations and Future Research

This was a single-site pilot, and the quantitative strand used descriptive observation without inferential testing. The 3 conditions were sequential and conducted on different dates, so temporal variation in visitor flow or other unmeasured contextual factors may have contributed to the differences. Counts represented anonymous passages, and repeat passages could not be identified. The 4 behavior indicators could overlap; their summed count per 100 passages does not measure the proportion of visitors who interacted, the depth or duration of engagement, or acceptance. Age category was not linked to individual behavior codes; therefore, age-related observations were limited to descriptive interpretation of the field notes.

The qualitative strand involved a small, staff-focused sample that differed from the observed visitor population. Children, parents or guardians, and other adult visitors were not interviewed. Although staff had direct exposure to the robot, their accounts reflect their own perceptions and cannot establish visitors’ perceived usefulness, ease of use, reasons for interacting, or acceptance. Because the datasets were not linked at the person level, integration cannot establish that staff-identified factors explain the observed behavioral differences. Exact staff age, gender, tenure, and job title distributions were not collected to protect the identity of employees. The study also did not directly measure child emotion, anxiety, trust, or satisfaction, although affect is central to social HRI [8,26]. These limitations restrict transferability and indicate that the 4-domain framework remains provisional.

Future research should combine in situ observation with ethically appropriate direct feedback from children, parents, adult visitors, and staff. Repeated or counterbalanced implementation periods and privacy-preserving person-level behavior coding would permit stronger comparison of contextual and age-related patterns. Longer follow-up should examine whether multilingual content, stronger interaction design, staff onboarding, explicit ownership, and maintenance arrangements support sustained use after novelty effects diminish.

Comparative work should also examine different robot-mediated tasks and settings. Wayfinding may require clear movement, stopping, and destination cues, while waiting time engagement may require age-appropriate content, audibility, and careful limits on distraction. Multisite studies could distinguish context-specific findings from more general implementation requirements and test whether the 4-domain framework remains useful across pediatric hospitals with different layouts, languages, staffing models, and patient populations.

### Conclusions

In this single-site descriptive pilot, observed visitor interaction with Temi V3 was higher under poster support and staff facilitation than at baseline. Staff perceived potential value in wayfinding, child engagement during waiting, and basic guidance, and they identified content, language coverage, reliability, support, and workflow fit as important implementation considerations.

These complementary findings informed a provisional mediated acceptance framework comprising role clarity, interaction affordance, social mediation, and organizational embedding. The framework is an interpretation of observed behavior and staff perspectives, not a demonstration of visitors’ acceptance or of the effects of specific support mechanisms. Direct feedback from children, parents or guardians, and other adult visitors, together with longer-term evaluation, is needed to assess these proposed conditions and their contribution to sustained use.

## Supplementary material

10.2196/95702 Multimedia Appendix 1 Supporting methodological and qualitative summary information.

## Abbreviations

HRI: human-robot interaction
RQ: research question
SAR: socially assistive robot
TAM: technology acceptance model
UTAUT: unified theory of acceptance and use of technology

## References

[1] Feil-Seifer D, Mataric MJ (2005). 9th International Conference on Rehabilitation Robotics (ICORR 2005).

[2] Henschel A, Laban G, Cross ES (2021). What makes a robot social? A review of social robots from science fiction to a home or hospital near you. Curr Robot Rep.

[3] González-González CS, Violant-Holz V, Gil-Iranzo RM (2021). Social robots in hospitals: a systematic review. Appl Sci.

[4] Holland J, Kingston L, McCarthy C (2021). Service robots in the healthcare sector. Robotics.

[5] Ragno L, Borboni A, Vannetti F, Amici C, Cusano N (2023). Application of social robots in healthcare: review on characteristics, requirements, technical solutions. Sensors (Basel).

[6] Silvera-Tawil D (2024). Robotics in healthcare: a survey. SN Comput Sci.

[7] Zhu T, Ahn HS, Broadbent E, MacDonald BA, Gasteiger N (2025). A deep dive into human-robot interaction in hospitals: scoping review on the services provided, engagement behaviours and interaction outcomes. Int J Soc Robot.

[8] Moerman CJ, van der Heide L, Heerink M (2019). Social robots to support children’s well-being under medical treatment: a systematic state-of-the-art review. J Child Health Care.

[9] Kabacińska K, Teng KA, Robillard JM (2025). Social robot interactions in a pediatric hospital setting: perspectives of children, parents, and healthcare providers. Multimodal Technol Interact.

[10] Papadopoulos I, Koulouglioti C, Lazzarino R, Ali S (2020). Enablers and barriers to the implementation of socially assistive humanoid robots in health and social care: a systematic review. BMJ Open.

[11] Bradwell HL, Aguiar Noury GE, Edwards KJ, Winnington R, Thill S, Jones RB (2021). Design recommendations for socially assistive robots for health and social care based on a large scale analysis of stakeholder positions. Health Policy Technol.

[12] Klebbe R, Klüber K, Dahms R, Onnasch L (2023). Caregivers’ perspectives on human–robot collaboration in inpatient elderly care settings. Machines.

[13] Aymerich-Franch L, Gómez E (2024). Public perception of socially assistive robots for healthcare in the EU: a large-scale survey. Comput Hum Behav Rep.

[14] Introducing temi robot V3 - in education. temi USA.

[15] Leoste J, Strömberg-Järvis K, Robal T, Marmor K, Kangur K, Rebane AM (2024). Testing scenarios for using telepresence robots in healthcare settings. Comput Struct Biotechnol J.

[16] Davis FD (1989). Perceived usefulness, perceived ease of use, and user acceptance of information technology. Manag Inf Syst Q.

[17] Venkatesh V, Morris MG, Davis GB, Davis FD (2003). User acceptance of information technology: toward a unified view. MIS Q.

[18] Heerink M, Kröse B, Evers V, Wielinga BJ (2010). Assessing acceptance of assistive social agent technology by older adults: the Almere model. Int J Soc Robot.

[19] Servaty R, Kersten A, Brukamp K, Möhler R, Mueller M (2020). Implementation of robotic devices in nursing care. Barriers and facilitators: an integrative review. BMJ Open.

[20] Ohneberg C, Stöbich N, Eberl I (2025). Possible applications and conditions for the development and integration of robotic assistance systems in the acute inpatient setting from the perspective of nursing professionals: a qualitative study. Int J Soc Robot.

[21] Creswell JW, Plano Clark VL (2017). Designing and Conducting Mixed Methods Research.

[22] Elo S, Kyngäs H (2008). The qualitative content analysis process. J Adv Nurs.

[23] Hsieh HF, Shannon SE (2005). Three approaches to qualitative content analysis. Qual Health Res.

[24] Braun V, Clarke V (2021). One size fits all? What counts as quality practice in (reflexive) thematic analysis?. Qual Res Psychol.

[25] Malterud K, Siersma VD, Guassora AD (2016). Sample size in qualitative interview studies: guided by information power. Qual Health Res.

[26] Stock-Homburg R (2022). Survey of emotions in human–robot interactions: perspectives from robotic psychology on 20 years of research. Int J Soc Robot.

