# Supplementary material for: Evaluating Human-Robot Interaction With a Socially Assistive Robot in Pediatric Health Care: Mixed Methods Pilot Study
Source: JMIR Nurs. 2026 Nov 6;9:e95702. doi: 10.2196/95702 (PMC13742777; doi:10.2196/95702)
Supplement: Supplementary file 1 [file nursing-v9-e95702-s001.docx]

Multimedia Appendix 1

# Supplementary staff-sample characteristics and qualitative-theme overview

This appendix presents supporting methodological and qualitative summary information.

*Table S1*. Available characteristics of the staff interview sample.

| **Sample characteristic** | **Available information** |
| --- | --- |
| Sample size | 12 health care professionals |
| Interview format | Four small focus-group sessions |
| Sampling approach | Purposive, criterion-based sampling |
| Inclusion criterion | Employment at the study site and direct exposure through use, observation, support, or deployment coordination |
| Outside the interview sample | Children, parents or guardians, other adult visitors, and staff without direct robot exposure |
| Role perspectives represented | Frontline clinical and nursing perspectives; coordination-level and senior organizational perspectives |
| Demographic detail available | Exact age, gender, tenure, and job-title counts were not retained in the deidentified analytic dataset |

Exact age, gender, tenure, and job-title counts are absent to protect the identity of the employees.

*Table S2*. Overview of the identified staff themes.

| **Theme** | **Summary of staff perspective** | **Relevance for acceptance** |
| --- | --- | --- |
| Role clarity and local usefulness | Staff wanted the robot to solve recognizable problems, especially wayfinding, room finding, and basic hospital instructions. | Perceived usefulness and role legitimacy |
| Value for children and families | The robot was seen as most useful when it engaged children during waiting or helped parents navigate. | Social value connected with practical benefit |
| Need for mediation and onboarding | Visitors and some staff did not fully know what the robot could do; prompting and explanation were often needed. | Social influence and learnability |
| Functional and technical limits | Participants cited speed, content, audibility, charging, multilingual gaps, and troubleshooting needs. | Ease of use and trust |
| Organizational ownership | Staff requested a designated person or unit for updates, support, and coordination with hospital routines. | Facilitating conditions and sustainability |
